# The case for integrated health and community literacy to achieve transformational community engagement and improved health outcomes: an inclusive approach to addressing rural and remote health inequities and community healthcare expectations

**DOI:** 10.1017/S1463423620000481

**Published:** 2020-12-02

**Authors:** Debra Jones, David Lyle, Lindy McAllister, Sue Randall, Robert Dyson, Danielle White, Aimee Smith, Denise Hampton, Mariah Goldsworthy, Alesha Rowe

**Affiliations:** 1 Director Primary Health Care, Broken Hill University Department of Rural Health, The University of Sydney, Broken Hill, Australia; 2 Head of Department, Broken Hill University Department of Rural Health, The University of Sydney, Broken Hill, Australia; 3 Professor Emerita, Faculty of Medicine and Health, The University of Sydney, Australia; 4 Senior Lecturer in Primary Health Care, The University of Sydney Susan Wakil School of Nursing and Midwifery, The University of Sydney, Camperdown, Australia; 5 Bachelor of Education, Networked Specialist Facilitator, New South Wales Department of Education, Parramatta, Australia; 6 Bachelor of Nursing, Nurse Academic, Broken Hill University Department of Rural Health, The University of Sydney, Broken Hill, Australia; 7 Bachelor of Social Work, Social Work Academic, Broken Hill University Department of Rural Health, The University of Sydney, Broken Hill, Australia; 8 Grad Certificate Indigenous Health Promotion, Aboriginal Program Coordinator, Broken Hill University Department of Rural Health, The University of Sydney, Broken Hill, Australia; 9 Grad Certificate Indigenous Health Promotion, Aboriginal Program Officer, Broken Hill University Department of Rural Health, The University of Sydney, Broken Hill, Australia; 10 Team Leader, Coomealla Health Aboriginal Corporation, Coomealla, Australia

**Keywords:** Australia, community engagement, community literacy, community literate, healthcare framework, health literacy, health executives, professional practice, rural and remote

## Abstract

**Context:**

Despite the substantial investment by Australian health authorities to improve the health of rural and remote communities, rural residents continue to experience health care access challenges and poorer health outcomes. Health literacy and community engagement are both considered critical in addressing these health inequities. However, the current focus on health literacy can place undue burdens of responsibility for healthcare on individuals from disadvantaged communities whilst not taking due account of broader community needs and healthcare expectations. This can also marginalize the influence of community solidarity and mobilization in effecting healthcare improvements.

**Objective:**

The objective is to present a conceptual framework that describes community literacy, its alignment with health literacy, and its relationship to concepts of community engaged healthcare.

**Findings:**

Community literacy aims to integrate community knowledge, skills and resources into the design, delivery and adaptation of healthcare policies, and services at regional and local levels, with the provision of primary, secondary, and tertiary healthcare that aligns to individual community contexts. A set of principles is proposed to support the development of community literacy. Three levels of community literacy education for health personnel have been described that align with those applied to health literacy for consumers. It is proposed that community literacy education can facilitate transformational community engagement. Skills acquired by health personnel from senior executives to frontline clinical staff, can also lead to enhanced opportunities to promote health literacy for individuals.

**Conclusions:**

The integration of health and community literacy provides a holistic framework that has the potential to effectively respond to the diversity of rural and remote Australian communities and their healthcare needs and expectations. Further research is required to develop, validate, and evaluate the three levels of community literacy education and alignment to health policy, prior to promoting its uptake more widely.

## Introduction

Despite substantial investments by Australian health authorities to improve the health of rural and remote communities (Humphreys and Wakerman, 2009; Standing Council on Health [SCoH], 2012; Health Workforce Australia [HWA], 2013), rural residents continue to experience healthcare access challenges and poorer health outcomes (SCoH, 2012). Similar findings are reported in Canada and the United States (Sibley and Weiner, 2011; Douthit *et al*., 2015). Increasingly, health authorities are using consumer education strategies to enhance the health literacy of individuals, promote individual capacity to access services needed, and ensure the provision of safe, quality care (WHO, 2009; Australian Commission on Safety and Quality in Health Care [ACSQHC], 2014). Whilst acknowledging the importance of health literacy, a focus on effecting individual behavior change can place undue burdens of responsibility on individuals for their own healthcare whilst not taking due account of broader community needs and healthcare expectations. This can also marginalize the influence of community solidarity and mobilization in effecting healthcare improvements.

Rural and remote populations are affected by complex geographical, educational, socio-economic, and health challenges that detrimentally impact on services and health outcomes (Humphreys and Wakerman, 2009; SCoH, 2012; HWA, 2013). Sallis, Owen, and Fisher (2008) cautioned that educating people to make healthy lifestyle and health promoting choices, in environments that are not supportive, can frequently result in weak and short-term effects.

The authors propose that there is a need to rethink how healthcare organizations and health personnel engage with communities with the goal of working as equal partners to establish and maintain supportive healthcare environments (Bowen, Newenham-Kahindi and Heremans, 2010). To achieve this, investments would be required that focus on the attainment of a critical level of community literacy in health personnel, inclusive of senior executives and frontline clinical and professional staff, in parallel with the continued development of health literacy in consumers.

**Figure 1.**
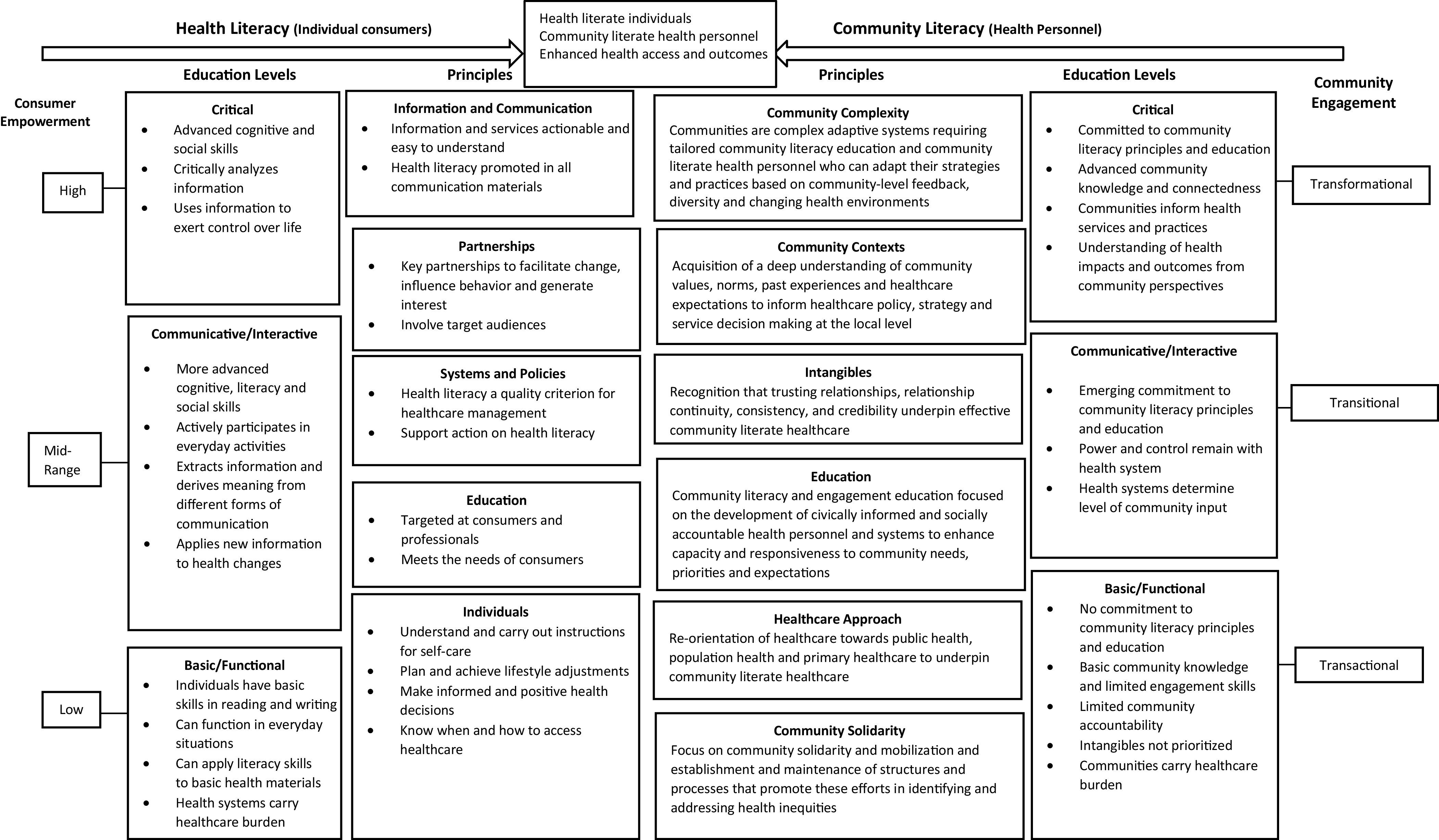
An integrated health literacy and community literacy healthcare framework.

The objective of this paper is to present a conceptual framework that describes community literacy, its alignment with health literacy, and their relationship to concepts of community engaged healthcare (Nutbeam, 2000; WHO, 2009; ACSQHC, 2014). The framework is informed by theory and literature concerning healthcare complexity (Hunter and Franken, 2012; Hawe, 2015), health systems (Gilson *et al*., 2007; Sallis *et al*., 2008; Hunter and Franken, 2012), community engagement (Bowen *et al*., 2010; Centers for Disease Control [CDC], 2011; Hyett *et al*., 2014), and rural and remote healthcare (Humphreys and Wakerman, 2009; Hyett *et al*., 2014). An integrated health and community literacy conceptual framework and model, a visual representation of the framework (Green, 2014), is presented to support concept interpretation and concept alignment with health literacy and rural and remote healthcare discourses. The framework is organized around health and community literacy principles, draws parallels between existing health literacy theory and education levels (Nutbeam, 2000) and proposed community literacy education levels, and health literacy and community engaged healthcare acquisition levels (see Figure 1).

Community literacy can contribute to the attainment of transformational community engagement. Insights acquired by health personnel can result in a greater capacity to engage, adapt, and influence primary, secondary, and tertiary healthcare provision within the communities and regions they serve as well as to enhanced opportunities to promote health literacy. This integration would inform a more equitable re-balancing of healthcare burdens between individuals with varying degrees of health literacy, and health personnel that have a deep understanding of their communities.

Community engagement occurs across a continuum from transactional engagement (communities as passive recipients of health care information); transitional engagement (where interactions are two-way between health authorities and the community; however communities are not considered equal partners in decision-making); and transformational engagement (where communities are considered equal partners with shared decision-making responsibilities) (Bowen *et al*., 2010). Although transformational engagement is best placed to positively impact on health outcomes, this level of engagement is the most difficult to achieve and the least researched (Adamson, 2010; Chia, 2011). It is envisaged that the community literacy framework can contribute to addressing this difficulty and inform research approaches.

## Defining health literacy

Health literacy is ‘the degree to which individuals have the capacity to obtain, process, and understand basic health information and services needed to make appropriate health decisions’ (US Department of Health and Human Services, 2010). Adequate health literacy is perceived to enhance an individual’s capacity to take responsibility for their own health and the health of their family (Sørensen *et al*., 2012). The World Health Organization (1998: 10) stated that health literacy represents:

The cognitive and social skills which determine the motivation and ability of individuals to gain access to, understand and use information in ways which promote and maintain good health … By improving people’s access to health information and their capacity to use it effectively, health literacy is critical to empowerment.

Health literacy principles described in Figure 1 include multi-pronged strategies in consumer acquisition of health knowledge and skills, focusing on; (1) information and communication, with the provision of information that is actionable, easy to understand, and embeds health literacy in all consumer communication; (2) partnerships, including target audiences and key stakeholders that can facilitate individual behavior change; (3) systems and policies that embed health literacy as a quality criterion for healthcare provision; (4) education targeting individuals and health professionals, ensuring consumer needs are met; and (5) individual consumer characteristics, including capacity to understand and act on instructions for self-care, make positive health decisions, and to know when and how to access services (Nutbeam, 2000; WHO, 2009; ACSQHC, 2014).

The ACSQHC (2014: 2) National Statement on Health Literacy proposed that health professionals should deliver consumer-targeted education to enhance the ‘motivation and capacity of a person to access, understand, and apply information to make effective decisions about health and health care’. The ACSQHC (p. 4) also suggested that health professionals should assume that ‘most people will have difficulty understanding and applying complex health information and concepts’. Limited health literacy is associated with: individuals who experience lower education and socio-economic status; riskier health choices; diminished capacity to self-manage health conditions; and increased hospitalizations (WHO, 2009; ACSQHC, 2014). These are characteristics frequently associated with rural and remote populations (Humphreys and Wakerman, 2009; SCoH, 2012).

### The challenge to health literacy education and acquisition in rural and remote Australian contexts

Disadvantaged populations, such as rural and remote Australian populations, can be challenged by poor health literacy (Cyril *et al*., 2015) that can impede effective healthcare utilization. In addition, disadvantaged communities are often confronted with higher risk factor burdens for diseases and limited awareness of health resources. In these situations, the use of community engagement and health literacy approaches that work among non-disadvantaged populations without modification can contribute to failures to achieve desired levels of engagement and health outcomes (Cyril *et al*., 2015). Cyril *et al*. (2015: 2) stated that ‘current evidence shows that disadvantaged populations are not adequately approached or effectively engaged in the efforts taken by service providers and health interventionists to improve their health’.

Despite efforts to improve health literacy, there remain multiple challenges confronting communities, health services and health personnel that limit progress towards enhanced service access and health improvements (Gilson *et al*., 2007; Sallis *et al*., 2008; Humphreys and Wakerman, 2009; Bowen, Newenham-Kahindi and Heremans, 2010; Hunter and Franken, 2012; SCoH, 2012; HWA, 2013; Hyett *et al*., 2014; Carey and Crammond, 2015; Hawe, 2015). The conceptual framework presented in this paper seeks to address these challenges and bridge the gap between the rhetoric and realities of community engagement and health literacy attainment experienced within rural and remote contexts. Poor understanding of unique community contexts, and health personnel–community networks and relationships can undermine engaged healthcare approaches (Lin, Smith and Fawkes, 2014).

Hawe (2015: 309) stated that complex health strategies, such as health literacy, can be influenced by the ‘effect of community-based solidarity’. However, community-based solidarity challenges the growing emphasis on individualized healthcare which is the focus of health literacy (Hunter and Franken, 2012) that links individual responsibility with self-sufficiency and consumer empowerment (WHO, 2009; ACSQHC, 2014; Lin, Smith and Fawkes, 2014). Whilst the health literacy literature acknowledges the importance of consumer-orientated communication, there is a lack of evidence concerning consumer and community engagement in information design (Rowlands *et al*., 2015). Furthermore, the lack of health service investment in strategies to address the social determinants of health through community-engaged healthcare provision can exacerbate service inequities and contribute to individual and community disempowerment (Crondahl and Karlsson, 2016). Hunter and Franken (2012: 25) stated that:

As attention to health literacy grows as an area for policy intervention, policy discourse continues to draw on skills deficit and patient compliance, buttressed by the dominant political discourse of individual responsibility.

Rural and remote Australian communities can be the recipients of healthcare models and strategies that are focused on standardization, individualization, curative, and hospital-based interventions. The WHO (2008: 12) stated that ‘the rural poor are increasingly confronted with the progressive fragmentation of their health services, as “selective” or “vertical” approaches focus on individual disease control programmes and projects’. A lack of insight into unique community contexts, limited health system–community networks and relationships can further detract from these healthcare approaches (Lin, Smith and Fawkes, 2014). Health professionals can be poorly prepared for practice beyond hospitals and patient bedsides (HWA, 2013). Attempts to enhance individual health literacy in these settings can undermine health service and health professional credibility, contributing to community cynicism towards healthcare provision (Jones, 2017).

One way of tackling rural and remote health inequities is to engage communities in service redesign to address local needs (Hyett *et al*., 2014). However, the co-design of healthcare with communities can disrupt the privileging of expert knowledge and the rules that inform how health personnel interact with care recipients (Dunston *et al*., 2009). Furthermore, effective community engagement is influenced by trusting relationships between health personnel and communities. Trusting relationships facilitate collective action offering ‘an alternative approach to the economic individualism that has driven public policy analysis in recent decades’ (Gilson *et al*., 2007: 1453). Trusting relationships need to be underpinned by continuity of health personnel–community relationships, ethical commitments and social accountability (Frenk *et al*., 2010). Trust can be adversely affected by the frequent turnover of health personnel and the subsequent failures to engage and respond to community identified health needs (Humphreys and Wakerman, 2009; HWA, 2013).

A re-focusing of healthcare is required if we are to address health inequities by empowering communities and providing services that are responsive to these contexts. This includes greater investments in public health, population health, and primary healthcare (WHO, 2008; Humphreys and Wakerman, 2009; Lin, Smith and Fawkes, 2014). Healthcare strategies must be fit-for-context, accounting for the diverse geographical, social, economic and cultural contexts of rural and remote communities (Humphreys and Wakerman, 2009). When health personnel acquire a deeper understanding of the communities in which they are located, they are better positioned to address inequities and contribute to improvements in health behaviors, public health planning, service access, and health literacy outcomes (Cyril *et al*., 2015).

## Community literacy

Community literacy enables health personnel to appreciate community healthcare experiences and to access community expertise to better understand, interpret and align healthcare to community needs and expectations. This includes the incorporation of community knowledge, skills and resources into the design, delivery and adaptation of rural and remote healthcare policies, strategies and services. Commitment to, and investment in, community engagement and literacy education would promote community responsive, intelligent and tailored approaches to service provision and professional practice, with the potential to further enhance health literacy investments and impacts.

The ACQSHC (2014) statements on health literacy were used to guide the development of principles that support the development of community literacy. This approach acknowledges that health personnel can experience difficulties in understanding the complexity and diversity of rural and remote communities, their values and norms, and the resultant struggles that can be confronted in aligning healthcare and practices to community needs, contexts, and expectations.

We propose six principles that underpin community literacy and its contribution to the attainment of transformational community engagement. These principles are:

1. an appreciation that communities are complex requiring tailored responses that will evolve, especially as communities and healthcare environments change (Gilson *et al*., 2007; Hawe, 2015);
2. knowledge of community contexts, including a deep understanding of community values, norms, past experiences and healthcare expectations, to inform healthcare policy, strategy, and service decision-making at the local level (Hawe, 2015);
3. attention to intangibles, including a recognition that trusting relationships, relationship continuity, consistency, and credibility, underpin effective community engagement (Jones *et al*., 2018);
4. delivery of community literacy and engagement education focused on the development of civically informed and socially-accountable health personnel and systems to enhance capacity and responsiveness to community needs, priorities, and expectations;
5. willingness to adjust healthcare approaches, including a re-orientation of healthcare towards primary health care, population health, and public health to achieve improved health outcomes (WHO, 2008; Humphreys and Wakerman, 2009; SCoH, 2012; HWA 2013); and
6. focus on community solidarity and mobilisation and the establishment and maintenance of structures and processes that promote these efforts in identifying and addressing health inequities (Hawe, 2015; Jones *et al*., 2018).

### Health and community literacy education and acquisition levels

Nutbeam (2000) described three levels of health literacy education for consumers; (1) basic/functional health literacy, (2) communicative/interactive health literacy, and (3) critical health literacy. At the basic/functional level, traditional approaches to the communication of information on health risks and health system navigation are delivered to consumers. This approach to education has limited goals focused on enhancing consumer knowledge of health risks and health services; and strategies do not invite ‘interactive communication, nor do they foster skills development and autonomy’ (Nutbeam, 2000: 265). At the communicative/interactive level, the focus is on the development of personal skills within supportive environments. Strategies are directed towards improving individual capacity to ‘act independently on knowledge, specifically to improving motivation and self-confidence to act on advice received’ (Nutbeam, 2000: 265). In contrast, the critical education level supports ‘the communication of information, and development of skills which investigate the political feasibility and organizational possibilities of various forms of action to address social, economic, and environmental determinants of health’ (Nutbeam, 2000: 265), leading to the attainment of a high level of health literacy.

These skill levels can be transposed to community literacy development, education, and their alignment to community engagement. At the basic/functional level of community literacy development and education, health personnel have basic community knowledge and limited accountability and incentives to engage with communities in the identification of their health needs and solutions. Important intangibles, such as trusting relationships, relationship continuity, consistency, and credibility are not prioritized by healthcare organizations nor health personnel, reflecting characteristics associated with transactional engagement (Bowen, Newenham-Kahindi and Heremans, 2010). At this basic level, individuals and consumers are subjected to undue burdens of healthcare responsibility.

At the communicative/interactive level, organizations and health personnel act to involve communities in healthcare decision-making. However, power and control remain with the health system, determining to what extent community knowledge, feedback, experiences, and expectations inform healthcare reform, decision-making, and/or re-orientation. This reflects transitional community engagement characteristics (Bowen, Newenham-Kahindi and Heremans, 2010) and what the authors’ term mid-range community engaged healthcare.

At the critical education level, health personnel have advanced community insight and connectedness that enables them to critically analyze community identified health needs and solutions, understand the impacts and outcomes of healthcare decision-making and service delivery from the perspective of community members, and the ability to use this information to inform the adaptation and re-orientation of health services and practices, reflecting transformational engagement (Bowen, Newenham-Kahindi and Heremans, 2010).

## Discussion

The goal of health systems is to enhance healthcare service accessibility and the health outcomes of populations (WHO, 2008; 2009; SCoH, 2012; ACSQHC, 2014). The development of community literacy in health personnel, and adoption of community literacy principles, aligns to the broad consensus on what is needed to address health inequities and meet the diverse needs of communities (Gilson *et al*., 2007, WHO, 2008; Humphreys and Wakerman, 2009; Frenk *et al*., 2010; Hunter and Franken 2012; SCoH, 2012; HWA, 2013; Carey and Crammond, 2015; Hawe, 2015). This approach also has resonance with the call for health literacy to go beyond concepts of consumer education and individual behavior change. The WHO (2009) stated that health education should aim to influence not only individual lifestyle decisions but also to raise awareness of the social determinants of health and encourage individual and collective action to modify these determinants.

The integrated health and community literacy framework presented in this paper could contribute to informing how health literacy extends beyond concepts of consumer education and individual behavior change through supported community literacy education for health personnel and the attainment of transformational community engagement. The concepts of health and community literacy are not mutually exclusive; they are intrinsically linked and mutually-reinforcing. The alignment of health literate individuals and consumers with community literate health personnel in some of Australia’s most marginalized communities should position those communities and health services to better address their health inequities, enhance health service access, and improve health outcomes.

We propose the inclusion of community literacy as a critical concept in rural health policy and practice to guide the education of current and future health personnel and the development of health system capacity to engage with communities as equal partners in building and sustaining supportive health care environments. Transitioning community literacy from a concept into practice requires further discussion within the healthcare sector, the development of educational resources, and the inclusion of community voices to determine its acceptability and relevance within rural and remote Australian contexts. Its generalizability to larger regional and metropolitan contexts could also be considered. Further research will also be required to better understand how health services engage with rural and remote communities and to evaluate the processes, impacts, and outcomes of the adoption of the community literacy concept (Wolff and Frank, 2005), education and practice on health personnel, health services and communities.

## Conclusion

The re-balancing of responsibility for addressing healthcare access and service inequalities between individuals and communities on one hand, and health care organizations and personnel on the other, is a complex process. To this end, investment in community literacy education and development for health personnel is proposed. The integration of health and community literacy may provide a holistic framework that enables rural and remote communities to achieve substantive and sustainable health improvements. The authors acknowledge the diversity of rural and remote Australian communities and their healthcare needs and expectations, in response this framework may require further refinement to better reflect individual community contexts and to promote its uptake more widely.
